# A nanoporous interferometric micro-sensor for biomedical detection of volatile sulphur compounds

**DOI:** 10.1186/1556-276X-6-634

**Published:** 2011-12-16

**Authors:** Tushar Kumeria, Luke Parkinson, Dusan Losic

**Affiliations:** 1 Ian Wark Research Institute, University of South Australia, Mawson Lakes Boulevard, Adelaide, SA 5095, Australia

**Keywords:** nanoporous alumina, reflectometric interference spectroscopy, volatile sulphur compounds, hydrogen sulphide sensor, oral malodour

## Abstract

This work presents the use of nanoporous anodic aluminium oxide [AAO] for reflective interferometric sensing of volatile sulphur compounds and hydrogen sulphide [H_2_S] gas. Detection is based on changes of the interference signal from AAO porous layer as a result of specific adsorption of gas molecules with sulphur functional groups on a gold-coated surface. A nanoporous AAO sensing platform with optimised pore diameters (30 nm) and length (4 µm) was fabricated using a two-step anodization process in 0.3 M oxalic, followed by coating with a thin gold film (8 nm). The AAO is assembled in a specially designed microfluidic chip supported with a miniature fibre optic system that is able to measure changes of reflective interference signal (Fabry-Perrot fringes). When the sensor is exposed to a small concentration of H_2_S gas, the interference signal showed a concentration-dependent wavelength shifting of the Fabry-Perot interference fringe spectrum, as a result of the adsorption of H_2_S molecules on the Au surface and changes in the refractive index of the AAO. A practical biomedical application of reflectometric interference spectroscopy [RIfS] Au-AAO sensor for malodour measurement was successfully shown. The RIfS method based on a nanoporous AAO platform is simple, easy to miniaturise, inexpensive and has great potential for development of gas sensing devices for a range of medical and environmental applications.

## Introduction

Hydrogen sulphide [H_2_S] is a colourless, corrosive, flammable and highly toxic gas commonly known through its foul odor of rotten eggs. It can be produced in sewage by bacterial breakdown, in coal mines and in the oil, chemical and natural gas industries [1]. As an extremely toxic gas, its early detection is crucial to protect people from deadly exposures (>250 ppm) [2]. However, recent studies showed that at lower concentrations, H_2_S has important biological functions [3]. Micromolar levels of H_2_S have been observed in human tissues (brain and blood) suggesting that H_2_S is a constituent of cells, but its broader biological role is still not well understood [4]. One of the reasons for our poor understanding is the lack of sensitive and specific analytical methods for real-time measurements of H_2_S in a complex biological environment. An oral malodour with major presence of H_2_S that arises from bacterial metabolism of amino acids and proteins is another example of biomedical determination of H_2_S that can be used for diagnosis of specific diseases [5].

Oral malodour, also known as halitosis or bad breath, is largely caused by volatile sulphur compounds [VSCs], which are produced due to bacterial degradation of proteins present in the oral cavity [5]. In most cases, oral malodour originates as the result of microbial metabolism and degradation of proteins, especially those that contain cysteine and methionine, or peptides and aminoacids that are present in the salivary/gingival crevicular fluid or in food that is retained on the teeth. It has been previously reported that VSCs, such as hydrogen sulphide (which accounts for 80% of oral VSCs), methyl mercaptan, dimethyl sulphide and allyl mercaptan, are the major gases associated with unpleasant oral malodour [6,7].

Diagnosis of oral malodour is conventionally performed organoleptically by a trained expert [8]. However, such measurements are obviously variable and quantitatively limited [8]. Several analytical methods have been devised for detection of VSCs including gas chromatography, high performance liquid chromatography, colorimetric, UV-Visible and fluorescence spectrophotometry, electrochemical (amperometric and potentiometric) methods and volumetric titrations [9-11]. However, these methods are time-consuming or require expensive equipment, skilled operators, often require a large volume of sample and cannot be used for real-time measurements. Hence, development of new methods to address these limitations for the biomedical measurement of H_2_S is urgently required. Optical methods are particularly attractive due to their sensitivity, simplicity, low cost, potential for *in-situ *measurement and ease of miniaturisation.

Reflectometric interference spectroscopy [RIfS], based on Fabry-Perot thin polymer film interference, has been effectively explored over the last two decades, mainly by the Gauglitz group, for sensing and biosensing applications including gases, hydrocarbons, herbicides, proteins and DNA [12,13]. Studies by MJ Sailor's group showed that nanoporous structures such as porous silicon and porous anodic aluminium oxide [AAO] offer superior RIfS properties for chemical and biological sensing in comparison with thin polymer films [14-16]. The detection method is based on the reflection of white light at the top and bottom of porous structures, which generates a characteristic interference pattern with Fabry-Perot fringes [14]. Binding of the molecular species on the pore surface induces changes of refractive index and wavelength shifts in the fringe pattern that can be easily detected and quantified [14]. The ultimate advantage of a nanoporous AAO platform, instead of planar polymer films previously used for RIfS sensing and biosensing, is in providing a unique three-dimensional morphology of pore structures and the flexibility to be modified with specific functional groups [17-19].

**Figure 1:**
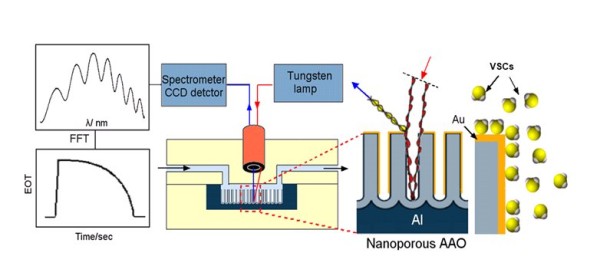
**Schematic of the RIfS device for gas sensing**. Scheme of detection of VSCs using nanoporous Au-AAO.

RIfS sensing using AAO was demonstrated for sensitive organic and biomolecular detection in aqueous solution, but the application for detection of gas molecules has not yet been considered. In this work, we present the first demonstration of nanoporous AAO for reflectometric interference H_2_S gas sensing and its practical application for malodour measurement. A schematic of our RIfS device with an AAO sensing platform assembled with a microchip device, light source, optical detection and data processing unit is shown in Figure 1. The nanoporous AAO layer is prepared on Al by electrochemical anodization, and it is composed of arrays of vertically aligned and highly organised (hexagonal pattern) pore structures with controllable pore diameters and pore length [20,21]. To achieve sensitivity and selectivity for H_2_S and VSC detection, the AAO surface was coated with gold which is known to have a good affinity with SH groups [22]. The gas detection is based on the changing of interference signal from the porous structure as a result of the adsorption of gas molecules on the gold-coated AAO surface. In this paper, we demonstrate the performance of this system for the practical application in VSC detection and real oral malodour monitoring.

## Experimental section

### Materials

Aluminium foil (0.1 mm, 99.997%) was supplied by Alfa Aesar (Ward Hill, MA, USA). Oxalic acid (Chem Supply, Pty Ltd, Adelaide, South Australia, Australia), chromium trioxide (Mallinckrodt, Inc., Miami, FL, USA), phosphoric acid (85%, BDH, VWR International Ltd., Poole Dorset, UK) and Na_2_S (Sigma-Aldrich Pty. Ltd., Sydney, Australia) were used as supplied. Standard gas concentrations for H_2_S measurements were prepared using a calibration of H_2_S gas mixture in air (BOC, Sydney, Australia) or by gas generated from Na_2_S in phosphate buffer and mixed with air. High purity water was used for all solutions preparation, as produced by sequential treatments of reverse osmosis, and a final filtering step through a 0.22-µm filter.

### Preparation of nanoporous AAO

Nanoporous AAO was prepared by a two-step anodization process using 0.3 M oxalic acid as electrolyte at 0°C as previously described [20,21,23]. The first anodized layer of porous alumina was prepared at a voltage of 60 to 80 V and then removed using an oxide removal solution (0.2 M chromium trioxide and 0.4 M phosphoric acid). Final anodization was carried out at 60 V for 10 min in order to prepare AAO with optimal pore diameters, inter-pore distances and length.

### Surface modification and structural characterisation of prepared AAO

The coating of ultra-thin metal films Au onto AAO (Au-AAO) was performed by metal vapour deposition (Emitech K975X, Quorum Technologies, Ashford, UK). The thickness of deposited films was approximately 8 nm and controlled by the film thickness monitor. The pore diameters and the thickness of the AAO porous film were determined by scanning electron microscopy [SEM] (FEI Quanta 450, FEI Company, Hillsboro, OR, USA). For cross-sectional SEM imaging, free-standing AAO substrates were prepared by removing the underlying Al. AAO samples were coated with a 5-nm Pt layer prior to SEM measurements.

### Fabrication and assembly microchip sensing device

**Figure 2:**
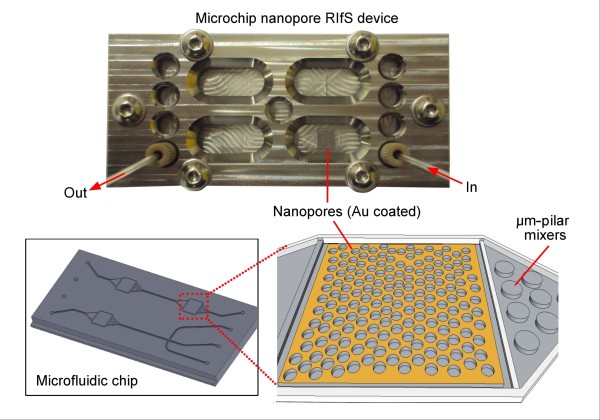
**The microfluidic nanoporous reflective interferometric RIfS device**. Photo with schematic representations of the embossing stamp and integrated AAO sensing platform within the microfluidic structure. Top photo shows the nanoporous microchip RIfS device, including the PMMA base chip bearing, the embossed microfluidic structure and the bondless device clamp. An enhanced view of the microstructures showing the position of nanoporous alumina and the micro-pillar mixer is clearly presented (bottom right).

To enable the facile integration of multiple AAO nanoporous sensor substrates to the microfluidic device, an unbonded microfluidic structure was fabricated in two reusable halves and sealed during use by fixing in a bondless microfluidic device clamp for hybrid materials (ANFF-SA, South Australia, Australia). The microstructures were formed in solid poly(methyl methacrylate) [PMMA] by the hot-embossing process using a brass stamp, machined by CNC micromachining (Supermill-2M, KIRA Corporation, Nishio, Aichi, Japan). The microfluidic structure integrated two channels (single inlet-single outlet and triple inlet-single outlet) with simple mixers, which allow even fluid or gas delivery to a cavity that accommodates the 5 × 5 mm Au-AAO sensing platform (Figure 2). The microstructures were replicated into the PMMA substrate by embossing under 4.3 MPa at 130°C using a hot embosser-substrate bonder (520-HE, EVG, St. Florian, Austria). Clamping of a Pyrex^® ^(Corning Inc., Corning, NY, USA) lid to the PMMA microfluidic structure sealed all channels. The bondless device clamp also facilitated the integration of the spectrophotometer. Figure 2 shows a photo of the nanoporous microchip RIfS device, including the PMMA base chip bearing the embossed microfluidic structure and the bondless device clamp (top). An enhanced view of the microstructures showing the position of nanoporous alumina and the micro-pillar mixer is clearly presented (bottom right).

### Optical setup for reflective interference measurements

Optical RIfS measurements were performed using a micro fibre optic spectrometer (Jaz-Ocean Optics, Inc., Dunedin, FL, USA). A bifurcated optical fibre with its one trunk illuminated by a tungsten lamp carried the light to the probe, and the reflected light was collected by the same probe and fed to the other trunk of the optical fibre, which at the end fed the reflected light to the spectrometer. The spot size of the light from the probe onto the AAO surface was kept around 2 mm in diameter, and all the reflective interference data were collected at a spectral range of 400 to 900 nm from the AAO film. Effective optical thickness [EOT] can be obtained by applying fast Fourier transform to the interference spectra. Fast Fourier transform from the Igor Pro (WaveMetrics, Inc., Portland, OR, USA) library was applied to finally obtain the EOT (2*n*_eff_*L *value in the Fabry-Perot interference fringe equation).

### Real-time malodour measurements

**Figure 3:**
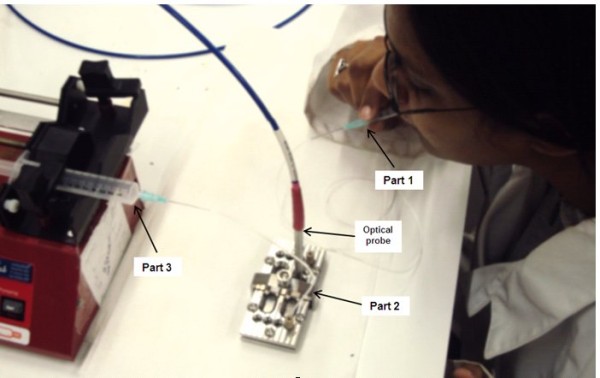
**The setup of real-time oral malodour measurements**. The use of RIFs sensing device showing the volunteer and the parts of sampling and sensing devices. The gas sampling was performed using three parts: a flexible straw connected to a neoprene tubing for suction (part 1), a tightly sealed microfluidic structure containing the AAO substrate (part 2), and a syringe pump for suction of a known volume of air (part 3).

The volunteers were subjected to an oral examination by a dentist, and only those with healthy oral hygiene were selected for the study. Three volunteers (two males and one female, age 20 to 30 years old) were examined. The volunteers were required to refrain from consumption of hot/cold beverages for at least 2 h before the gas sampling and breath measurements. The gas sampling was performed using three parts: a flexible straw connected to a neoprene tubing for suction (part 1), a tightly sealed microfluidic structure containing the AAO substrate (part 2), and a syringe pump for suction of a known volume of air (part 3) as shown in Figure 3. Before collection of a breath sample, the volunteers were asked to keep their mouth closed for 5 min. They were then instructed to insert the straw into their mouth, position the tip of the straw close to the middle of their tongue without touching it (to prevent entry of saliva) and hold it in position by closing their lips on the straw. Once the straw was positioned, the pump was operated at the rate of 250 µL/min for 3 min, drawing a total of 750 µL of air which was passed over the Au-AAO sensing platform. Prior to introduction of air from the patient's mouth, a stable clean-air baseline was established after 2 min of flow. After finishing the measurement, H_2_S-free air was again introduced at the same rate.

## Results and discussion

### Structural characterisation of prepared AAO

**Figure 4:**
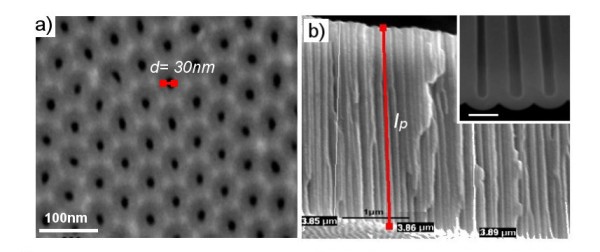
**SEM images of AAO pore structures used as sensing platform**. (**a**) The top AAO surface with ordered pores and (**b**) cross-sectional image showing vertically aligned pore structures. The bottom part of the pore structures with barrier layer surface is shown on the inset.

SEM images of the nanoporous AAO structure fabricated by anodization of Al in 0.3 M oxalic acid from the top surface and in cross-sectional view are shown in Figure 4, confirming the typical structure of AAO [20,21]. Images clearly represent (Figure 4a) the uniformly sized and regularly organized hexagonal pores and (Figure 4b) the cross-sectional view of a free-standing AAO structure with straight and vertically aligned pores with the bottom closed by a barrier oxide layer. The removal of Al from AAO is performed only for imaging purposes and for sensing; the Al layer is not removed. SEM images confirm that the pore diameter of the AAO is around 30 to 35 nm and the length to be around 4 µm which has been previously shown to generate optimal RIfS signal.

### Detection of H_2_S by RIfS Au-AAO sensor

**Figure 5:**
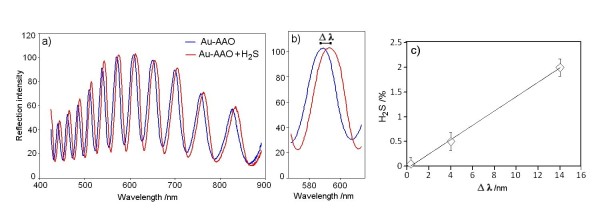
**Fabry-Perot interference response to sulphide gas**. (**a, b**) Fabry-Perot interference spectrum before and after exposure to hydrogen sulphide gas obtained from gold-coated porous alumina (Au-AAO) showing a shift of fringe pattern. (**c**) The gas concentration dependence graph.

To demonstrate the capability of the RIfS sensing device for VSC detection, in the initial experiment, the sensor was exposed to different H_2_S gas concentrations. H_2_S was specifically chosen as it is as a major component (80%) of oral malodour [24]. An interference spectrum was recorded before and after exposure of our Au-AAO sensor to H_2_S. Following the introduction of H2S, we observed a wavelength shift in our interference fringe spectrum (Figure 5a,b). The observed shift of wavelength and the corresponding change of EOT signal are attributed to a change of the porous refractive index of nanoporous AAO layer as a result of the adsorption of H_2_S molecules on the gold-coated surface. It was observed that these shifts (from 1 to 14 nm) or EOT changes correlated with the variation of H_2_S concentration in air between the values of 0% and 2%. This confirms the potential for this system to be applied not only for H_2_S detection, but also for measurements of H_2_S concentration. To check the selectivity of our sensing device, the sensor was exposed to different pure or mixed gases, such as hydrogen and air, with no significant changes observed in the interference pattern output. Exposure of the same AAO sensor without a gold coating to H_2_S gas also showed no significant change to the interference pattern output, which confirms the specific selectivity of the Au-AAO sensor for H_2_S molecules. These results are attributed to the specific affinity of Au to the S atoms, which underpins the function of the Au-AAO sensors for sulphur-containing compounds and potential RIfS oral malodour sensing devices.

### Real-time oral malodour measurements

**Figure 6:**
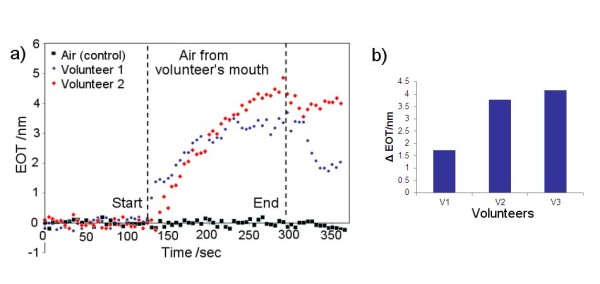
**Real-time measurement of total VSCs**. Result obtained from two volunteers showing increasing EOT signal when air from their mouth is introduced to the RIfS sensor. (**a**) The graph presents real-time optical response recorded as the EOT signal taken from mouth air of two volunteers and air control. (**b**) The graph shows the comparison of VSC measures for three volunteers measured with our system, clearly representing the ability of our device to distinguish oral hygiene conditions based on oral VSCs.

After demonstrating the ability of the RIfS system for detection of H_2_S, the performance of our device was examined for oral malodour analysis in three volunteers with normal oral hygiene. Figure 6a presents real-time optical response recorded as the EOT signal taken from mouth air of two volunteers and air control. A large increase of the EOT signal was observed when the mouth-air sample was introduced to the sensing device, in comparison with the EOT change observed following the introduction of clean air. Figure 6b shows the comparison of VSC measures for three volunteers measured with our system clearly representing the ability of our device to distinguish oral hygiene conditions based on oral VSCs. The results obtained from our system correlated well with organoleptic measurements of oral malodour from all the three subjected volunteers. It is well documented that H_2_S is the major (80%) volatile sulphur compound present in the breath, where about 20% corresponds to methyl mercaptan and dimethyl sulphide [10]. As all these sulphur compounds have HS groups with a strong affinity for the gold surface, we conclude that the observed signal yields a value which represents the concentration of total VSCs. An average concentration of total sulphur compounds in the malodour of healthy individuals is between 0.2 to 0.4 µg/L [24]. Detection of VSCs within these limits by the Au-AAO sensor confirms the high sensitivity of this sensor and its suitability for making such measurements [24]. By changing the surface chemistry of AAO with self-assembled monolayers with functional groups which are specifically sensitive for binding targeting molecules including gases, metal ions, organic molecules or even cells, this method can be applied for a broad range of analytical applications. A comparative study with gas chromatographic analysis will be performed to evaluate more precisely the performance of our Au-AAO sensor for malodour measurements and potential clinical applications.

## Conclusion

In conclusion, nanoporous AAO RIfS sensing for the measurement of VSCs is demonstrated. Gold-coated AAO RiFS sensor was found to have an excellent sensitivity for H_2_S and VSCs based on the affinity of gold surface to binding HS groups. A practical biomedical application of the RIfS Au-AAO sensor for malodour measurement was also successfully proved. The RIfS gas detection method is generic, and the coating of AAO with other gas-sensitive films can be used for the detection of specific hazardous gases. The sensing device based on a nanoporous AAO platform is simple, easy to miniaturise, inexpensive and has great potential for the development of gas sensing devices for a range of medical and environmental applications.

## Competing interests

The authors declare that they have no competing interests.

## Authors' contributions

TK carried out all the experimental works including AAO preparation, Au deposition, SEM characterisation, assembly of RIfS sensing device, testing of sensing performance data processing and composition of the draft manuscript. LP was involved in designing and in the fabrication of the microfluidic system for the RIfS sensor. DL provided knowledge and supervision support for this study and wrote the final version of the paper. All authors read and approved the final manuscript.
